# Statistical Image Properties in Works from the Prinzhorn Collection of Artists with Schizophrenia

**DOI:** 10.3389/fpsyt.2017.00273

**Published:** 2017-12-11

**Authors:** Gudrun Maria Henemann, Anselm Brachmann, Christoph Redies

**Affiliations:** ^1^Experimental Aesthetics Group, Institute of Anatomy I, Jena University Hospital, University of Jena, Jena, Germany

**Keywords:** dementia praecox, experimental aesthetics, image analysis, edge orientations, self-similarity, Fourier spectrum

## Abstract

The Prinzhorn Collection preserves and exhibits thousands of visual artworks by patients who were diagnosed to suffer from mental disease. From this collection, we analyzed 1,256 images by 14 artists who were diagnosed with dementia praecox or schizophrenia. Six objective statistical properties that have been used previously to characterize visually aesthetic images were calculated. These properties reflect features of formal image composition, such as the complexity and distribution of oriented luminance gradients and edges, as well as Fourier spectral properties. Results for the artists with schizophrenia were compared to artworks from three public art collections of paintings and drawings that include highly acclaimed artworks as well as artworks of lesser artistic claim (control artworks). Many of the patients’ works did not differ from these control images. However, the artworks of 6 of the 14 artists with schizophrenia possess image properties that deviate from the range of values obtained for the control artworks. For example, the artworks of four of the patients are characterized by a relative dominance of specific edge orientations in their images (low first-order entropy of edge orientations). Three patients created artworks with a relatively high ratio of fine detail to coarse structure (high slope of the Fourier spectrum). In conclusion, the present exploratory study opens novel perspectives for the objective scientific investigation of visual artworks that were created by persons who suffer from schizophrenia.

## Introduction

The relation between mental dysfunction and creativity has fascinated artists and scientists alike. To quote the French artist Jean Dubuffet (1901–1985): “For me, insanity is super sanity. The normal is psychotic. Normal means lack of imagination, lack of creativity.” Indeed, many artists are believed to have suffered from psychiatric disorders, as discussed, for example, Edvard Munch (1) or Abstract Expressionist artists of the New York School (2). Recent studies revealed that there are links between schizophrenia and creativity, also regarding similar genetic roots (3–6). Nevertheless, it is not clear whether mental illness is a cause of creativity or only affects it.

There is a continuous spectrum from artists who suffered from schizophrenia to patients with schizophrenia who created art (7). For example, the artist Richard Dadd is suspected to have suffered from schizophrenia (8). Friedrich Schröder-Sonnenstern (1892–1982) and Adolf Wölfli (1864–1930) are examples of patients who were diagnosed with schizophrenia and started drawing while they spent time in a psychiatric institution; they are now considered important representatives of Outsider Art or Art Brut (9, 10).

Psychiatrist and art historian Hans Prinzhorn (1886–1933) was deeply interested in the art of the mentally ill (11). During his time at the Psychiatric Hospital of Heidelberg University (1919–1921), he established the now world-famous Prinzhorn Collection that comprised about 5,000 artworks at his time (7). The collection gradually expanded to 26,000 artworks, which are now preserved at Heidelberg University in a special museum that is dedicated to the works of artists who were residents of psychiatric institutions.^1^

Prinzhorn himself analyzed his collection extensively (7, 12). For example, in the artworks of his collection, he described a “horror vacui” (i.e., a tendency to fill every last corner of an image), ornamental patterns and repetitive elements, a choice of motifs neither with higher meaning nor according to the laws of nature, as well as a preference for religious or erotic content (12). Later, other scientists attempted to find stylistic elements that are specific for art by persons with schizophrenia, for example, the psychiatrists Leo Navratil (13) and Helmut Rennert (14). In their studies, they described specific image characteristics, such as repetitive motifs.

Some decades later, more structured analyses of paintings by the mentally ill were undertaken. For example, Hacking et al. analyzed color, brightness and intensity of color, line thickness, percentage of space covered, and emotional tone in 50 pictures painted by patients with diverse psychiatric backgrounds (15). This pilot study suggested that there might be formal differences in paintings according to diagnosis groups.

Recently, more sophisticated image analysis tools have become available to analyze visual artworks in a more objective way (16). For example, in a pilot study, Graham and Meng (17) determined the spatial frequency spectral properties of a total of 12 paintings by 5 artists with schizophrenia. They showed that their works contained a larger proportion of high spatial frequencies than control paintings by artists without schizophrenia. Forsythe et al. (18) determined age-indexed variations of the fractal dimension, a measure that relates to subjective complexity, in 2,092 works of art by seven artists who experienced either normal aging or neurodegenerative disease. The authors proposed that changes in the fractal dimension might be useful in the early detection of neurodegenerative processes.

**Figure 1:**
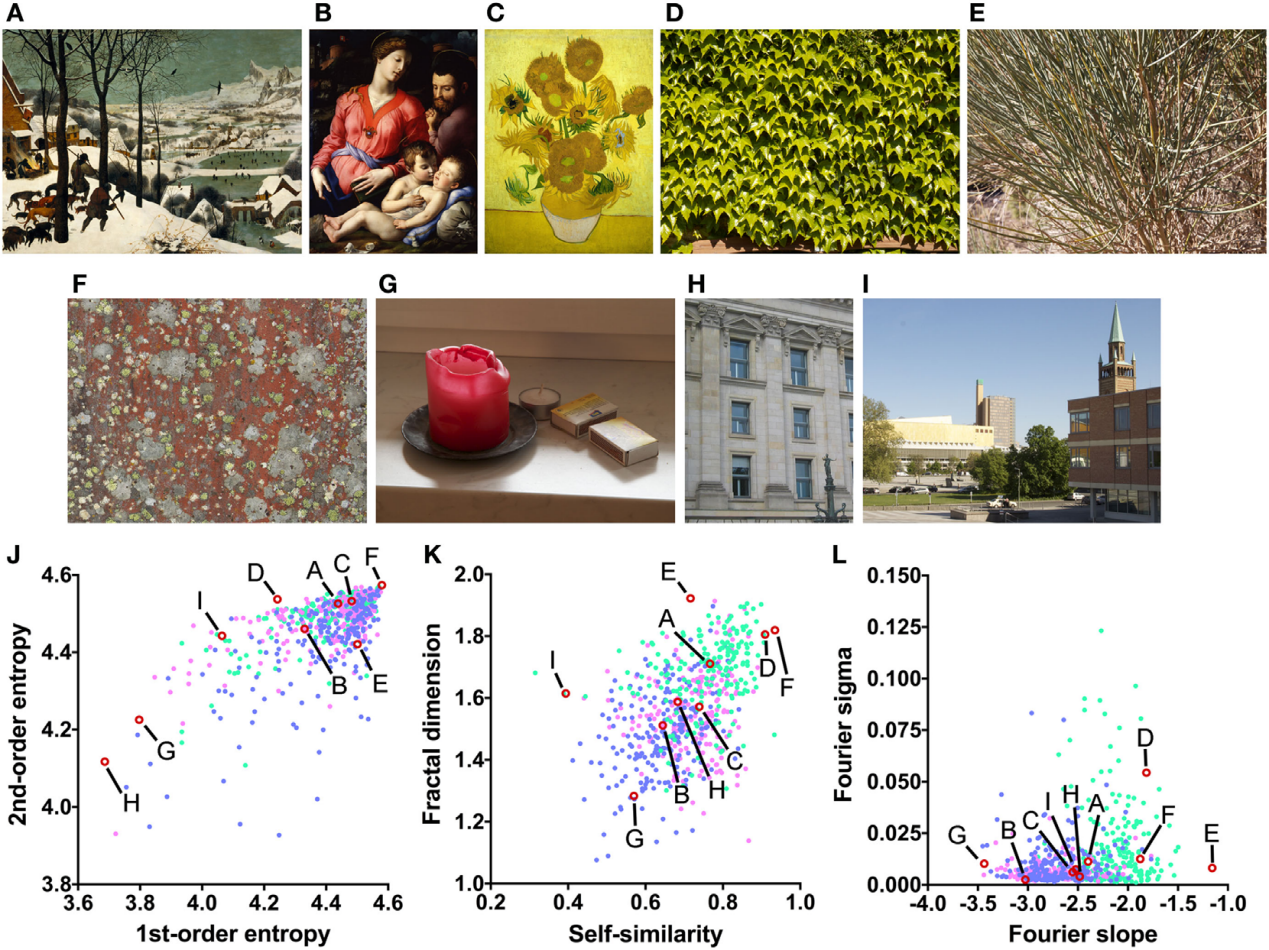
Examples or art and non-art images **(A–I)** and their image properties [**(J)** first-order and second-order entropy; **(K)** self-similarity and fractal dimension; **(L)** Fourier slope and sigma]. In the dot plots, each dot represents one image (*pink dots*, oil paintings; *green dots*, graphic art; *light blue dots, Bad Art*). The letters and the *red open circles* in **(J–L)** indicate the values of the exemplary images **(A–I)**. The paintings reproduced in **(A–C)** are in the public domain [**(A)** Pieter Brueghel the Elder, The Hunters in the Snow, 1565; **(B)** Angelo Bronzino, The Panciatichi Holy Family, about 1540; **(C)** Sunflowers, Vincent van Gogh, 1888].

While such studies suggest that it might be possible to find hints of mental disorders in an artist’s work, quantitative studies that apply objective measures to large sample of artworks are still scarce to date. In the present explorative study, we therefore use modern computational approaches to analyze statistical image properties in 1,256 artworks by 14 artists of the Prinzhorn Collection. The image properties have been used previously in the field of experimental aesthetics to characterize visual artworks (16). Exemplary real-world photographs that demonstrate the properties are shown in Figure 1.

Specifically, the following image properties were analyzed:

(1) *First-order entropy of edge orientations* (Figure 1J), which reflects how evenly luminance edge orientations in an image are distributed across all orientations (19). For example, the lichen growth pattern (Figure 1F) displays all orientations at a similar strength (high first-order entropy) while cardinal orientations predominate in the photographs of the building facade (Figure 1H; low first-order entropy).
(2) *Second-order entropy of edge orientations* (Figure 1J), a measure of how independent luminance edge orientations are across an image (19). For example, orientations are independent across images of many natural growth patterns, such as the patterns of lichen (Figure 1F) and leaves (Figure 1D; high second-order entropy), but they are spaced more regularly in building facades (Figure 1H; low second-order entropy).
(3) *Self-similarity* (Figure 1K), a measure of how similar the histograms of orientated gradients (HOGs (20)) are in parts of an image compared to the entire image (21). For example, self-similarity is high in the photograph of the leaves (Figure 1D), while it is low in the photograph of the urban scene (Figure 1I).
(4) The *fractal dimension* (Figure 1K), which relates to the subjective complexity of an image (22). For example, the photograph of the bush (Figure 1E) has a much higher fractal dimension (complexity) than the photograph of the candle (Figure 1G).
(5) The slope of a straight line fitted to log-log plots of radially averaged plots of Fourier spectral power (here called *Fourier slope*; Figure 1L); the slope indicates the relative strength of high spatial frequencies (i.e., fine detail) versus low spatial frequencies (i.e., coarse structure) of luminance changes in an image (23, 24). For example, the photograph of the bush (Figure 1E) contains a large amount of fine detail and little coarse structure (high Fourier slope). In the oil painting (Figure 1B) and the photograph of the candle (Figure 1G), coarse structure is relatively prominent when compared to the amount of fine structure (low Fourier slope).
(6) The deviation of the measured Fourier spectral power from the fitted straight line (here called *Fourier sigma*; Figure 1L). This measure indicates how well the Fourier spectrum can be fitted to a straight line. Most natural images (Figures 1A–C,E–J) have low values because the spectral amplitude decreases linearly with increasing spatial frequency in log-log plots (23–25). In Figure 1D, the spectrum deviates from the straight line because of the high amplitude of restricted spectral frequencies that reflect the regular periodicity of the leaves.

Previously, large subsets of traditional artworks (for examples, see Figures 1A–C) were shown to possess high first-order and second-order entropy of edge orientations (19), an intermediate to high self-similarity (26), and an intermediate fractal dimension (22) (Figure S1 in Supplementary Material). Furthermore, most natural images (25) as well as image of traditional artworks of Western and Eastern provenance (23, 24) possess Fourier spectra that decrease linearly with increasing spectral frequency, when radially averaged Fourier power is plotted in log-log space. The mean slopes range from around −2 for monochrome graphic art to −3 for oil paintings (23, 24). The Fourier sigma is low on average (23, 24). This result implies that the images share a fractal-like (scale-invariant) power spectrum. In contrast, some uncomfortable images possess Fourier spectra that deviate from straight lines (27, 28). For a more detailed description of the measures, see Section “Materials and Methods.”

To determine whether artworks by patients with schizophrenia possess properties similar to traditional art, we compared them to three control data sets of artworks that covered a wide spectrum of traditional art genres, subject matters, techniques, and artistic skills. First, many artworks by the patients were highly colored. Therefore, we analyzed a control data set of 1,629 mostly colored oil paintings of Western provenance from prestigious museums and art collections (21, 29). A control data set of colored works on paper, which would have been even closer in technique to the patients’ artworks, was not available to us. Second, a similarly diverse data set of 200 monochrome graphic artworks (24) was used as a control because many of the patients’ works were monochrome. Third, with notable exceptions, few of the artists from the Prinzhorn Collection had received formal artistic training (Table 1). Therefore, we included a control data set of 288 artworks from 2 museums that collect works by contemporary artists who possessed lower artistic skills in general [so-called *Bad Art* from the © Museum of Bad Art (acronym: MOBA) in Somerville, MA, USA, and the Official Bad Art Museum of Art (acronym: OBAMA) in Seattle, WA, USA (30)]. We assumed that only a few or none of the artists from the three control data sets suffered from mental illness.

**Table 1:** Overview of the 14 artists from the Prinzhorn Collection.

Name	Date	Profession	Artistic training	Diagnosis	Reference
Else Blankenhorn	1873–1920/21	None	Training in painting, photography and music	Dementia praecox, catatonia	Brand-Claussen (11); Brand-Claussen and Stephan (31)
Franz Karl Bühler	1864–1940	Artist, blacksmith	Professional	Dementia praecox	Brand-Claussen (11); Brand-Claussen and Stephan (31)
Elisabeth Faulhaber	1890–1921	Housemaid/servant	?	Schizophrenia (“Jugendirresein”)	^a^
Paul Goesch	1885–1940	Master builder and painter	Professional	Schizophrenia	Brand-Claussen (11); Brand-Claussen and Stephan (31)
Gustav Grube	?	“Confiseur”	?	?	^a^
Heinrich Hack	1869–?	Ffactory worker		Schizophrenia	^a^
Oskar Herzberg	1844–1917	Lithographer	Professional	Schizophrenia	^a^
August Klett	1866–1928	Wine merchant	None	Dementia praecox	Brand-Claussen (11)
Peter Meyer	1871/1872–1930	Innkeeper	None	Schizophrenia	^a^; Brand-Claussen (11); Brand-Claussen and Stephan (31); Prinzhorn (12)
August Natterer	1868–1933	Electrician	Professional	Schizophrenia	^a^; Brand-Claussen (11)
Joseph Schneller	1878–1943	Architectural draftsman	Professional	Schizophrenia	^a^
Oskar Voll	1876–1935	Tailor	Professional?	Schizophrenia, paranoia	^a^; Brand-Claussen and Stephan (31)
Clemens von Oertzen	1853–1919	Naval officer	None	Dementia paranoides	Brand-Claussen (11)
Frau von Zinoview	?	?	?	?	

*^a^Information kindly provided by Dr. Thomas Röske, Sammlung Prinzhorn*.

*?, unknown*.

We asked the following questions:

(1) Can systematic differences in the image properties between the works of the 14 individual artists with schizophrenia be found?
(2) Do the works of some artists with schizophrenia deviate from those of traditional Western artworks or *Bad Art*?
(3) If so, can the deviating properties be related to the subjective visual impact that some artworks of patients with schizophrenia have on the beholder?

## Materials and Methods

### Data Sets of Artwork Images

High-quality digitized reproductions of works by 14 artists who are featured in the Prinzhorn Collection were kindly provided by the curator of the collection, Dr. Thomas Röske. For biographic details of the artists and their diagnoses, see Table 1. Most of the artists were diagnosed with dementia praecox or schizophrenia. However, the diagnoses of two of the artists are unknown. Artworks with large artifacts (decolorations, tears, folds, etc.), as well as images with regular written text that covered more than one third of the image area, were excluded from the analysis. Images containing artistic calligraphy, such as found in the artworks by Heinrich Hack and Peter Meyer, were not excluded. If required, images were cropped to the borders of the artworks.

For reasons stated in Section “Introduction,” we used the following three data sets of art images for comparison:

(i) 1,629 images of paintings of Western provenance [JenAesthetics dataset (29, 32)]. The paintings were created by well-known artists of the 16th to 20th centuries (up to 1937) and were downloaded from artwork databases (e.g., Google Art Project in Wikimedia Commons). Within this data set, we also compared the patients’ artworks to a subset that comprised the 427 paintings from the time period 1880–1937. This subset of artworks was roughly contemporary to the patients’ work and was therefore presumably more similar in artistic style to the patients’ artworks than the artworks from other periods.
(ii) 200 images of graphic artworks, including etchings, drawings, woodcut prints, lithographs, etc. that were scanned from various art books (24). The range of artworks included in this data set was similar to the painting dataset.
(iii) 288 works of *Bad Art* from the MOBA and the OBAMA [see Introduction (30)]. The 244 MOBA images represented photographs of the original artworks and were kindly provided as digitized reproductions by the curator of the museum, Mr. Michael Frank. The 44 OBAMA images were downloaded from the official website of the museum.^2^ The artworks date from the late 20th century to the early 21st century. Only images of high resolution and without obvious visible artifacts (blurring, JPEG artifacts, reflections, etc.) were analyzed. Any frames around the images were digitally removed.

### Image Analysis

The calculation of the statistical image properties (see Introduction) followed previously published procedures. Briefly, we determined the following properties.

#### First-Order and Second-Order Entropy of Edge Orientations

The spatial distribution of the edge orientations across each image was studied by calculating the first-order and second-order Shannon entropies of the edge orientation histograms [for a detailed description of the method, see Ref. (19)]. First, we converted color images to grayscale images using the ITU-R-601-2 luma transform, which weights the color channels according to their perceived luminosity. Following downscaling of the original input images to a total size of 120,000 pixels while preserving the aspect ratio, edges were extracted by applying a set of 24 oriented Gabor filters, which represented one full rotation when combined. In our analysis, we included the 10,000 strongest responses and excluded responses within 15 pixels of the image border.

*First-order entropy* was defined as the entropy of the histogram that summed up the strength of all edge orientations across the entire image. First-order entropy is maximal (about 4.585 for 24 bins of orientations) if all orientations are represented at equal strength across an image.

*Second-order entropy* was determined by a pairwise comparison of all edge elements in an image (19). For each edge pair, we normalized the orientation of the first (reference) edge to be horizontal. Then, 1d histograms were generated for all distances *d* (500 bins) and radial directions α (48 bins) between the edge pairs, by summing up the relative orientations θ (24 bins across the full circle of orientations) between all edge pairs at each location *d*, α (here called θ histograms). The normalized θ histograms represent the weighted probability of observing, for any given reference edge, an edge with an orientation difference θ at a distance *d* and in direction α. As a measure of the uniformity of the θ histograms, the Shannon entropy *H*(*X*) of the histograms was calculated as follows:

(1) $$H(X)=-\overset{n}{\underset{i=1}{\sum }}p(x_{i})\cdot {\text{log}}_{2}p(x_{i}),$$

where *X* is the θ histogram at angle α and distance *d*. The theoretical entropy maximum for the 24 bins in the θ histogram is about 4.585. Entropy values close to this value indicate that the θ histogram is highly uniform, i.e., all orientations are about equally likely to occur relative to the orientation of the reference edge at an angle α and a distance *d*. In other words, higher entropy values mean that edge orientations are more independent of each other in an image. For less uniform histograms, where particular orientations are more prevalent than others, entropy is lower. Low values imply that the orientation of one edge in an image allows predicting the orientation of other edges in the image with an above-random probability. To simplify the results, we calculated entropy as a function of distance *d* by averaging second-order entropy across directions α and then averaging the values for the distance range between 20 and 80 pixels, as done previously (19). Note that, in general, second-order entropy can be high only if first-order entropy is also high, i.e., first-order and second-order entropy are not independent of each other. Second-order entropy is maximal if all relative orientations occur at equal strength in the histograms, that is, if the orientation of a given edge does not allow predicting the orientation of other edges in the image, i.e., if edge orientation is independent of each other across an image.

#### Self-Similarity of Gradient Orientations

Self-similarity was calculated with a method that was derived from the Pyramid Histogram of Oriented Gradients (PHOGs) descriptor (33), as described before (21, 34). Briefly, we transformed color images into the Lab color space and reduced the size of all images uniformly to 100,000 pixels by bicubic interpolation and isotropic scaling (34). We then calculated the PHOG descriptor by generating histograms of oriented luminance gradients [HOG features (20)] for each image at consecutive levels of an image pyramid (33). We obtained histograms for 16 equally sized bins that covered the full circle (360°) (26). To begin with, the HOG features were calculated at the ground level (level 0), that is, for the entire image. Then, the image was divided into four equally sized rectangles (level 1). HOG features were calculated for each of the four sections at this level. Each section at level 1 was again divided into four equally sized rectangles to generate the next level of the pyramid and so on. Thus, level 2 comprised 16 sections and level 3 contained 64 sections. We calculated the HOG features for each section at a given level.

Finally, the histograms at different levels of the pyramid were compared with the ground level histogram (21, 26) to obtain a measure of self-similarity. We calculated self-similarity as the mean value for levels 1–3 of the pyramid. Self-similarity is higher if the histograms at different levels of the pyramid are more similar to the histogram at the ground level. A value of 0 indicates minimal self-similarity, and a value close to 1 nearly complete self-similarity. A detailed description of the method can be found in the Appendix in the study by Braun et al. (34).

#### Fractal Dimension

We measured the fractal dimension of each image with the box-counting method (35). Because this method requires binarized images, we filtered each image by applying a canny-edge filter (36). Empty areas at the border of each image were cropped to avoid a distortion of the result. We then covered the cropped image by a mesh of equally sized squares (“boxes”). Note that some of the boxes contain part of the pattern, while others remain empty. We repeated this procedure for decreasing box sizes ϵ. With an increasingly finer mesh, the number of boxes that contain parts of the image becomes larger. We defined N(ϵ) as the number of boxes that are occupied by parts of the image in a mesh of box size ϵ. According to the power law relation $N(\epsilon )∼\epsilon^{-D}$, the box-counting dimension *D* can now be determined by measuring the slope of the line that fits the plot log(N(ϵ)) versus $\log\left(\frac{1}{\epsilon }\right)$ best.

Simple forms, such as dots, lines, or squares, have a fractal dimension that is equal to their Euclidean dimension. More sophisticated patterns, for example, complex curves in 2d space, have a fractal dimension between 1 (low complexity) and 2 (high complexity). Humans tend to prefer natural and artificial patterns with a fractal dimension between 1.3 and 1.5 (22), but individuals differ considerably in their preferred range (37).

#### Fourier Slope and Sigma

The slope of log-log plots of the radially averaged Fourier power spectrum was calculated as described previously (23–25). To obtain the Fourier spectrum of each image, we first padded images according to square ones by adding a uniform border that displayed a gray level equal to the mean gray level of the image. We then reduced all images to a size of 1,024 × 1,024 pixels by bicubic interpolation and isotropic scaling. A 2d power spectrum was obtained by discrete Fourier transformation. We converted the resulting 2d spectrum into a 1d spectrum by rotationally averaging power for each frequency. Power was then plotted in the log-log scale as a function of spatial frequency. Next, data points were binned at regular frequency intervals in the log-log plane. A least-squares fit of a straight line to the binned data was carried out in the frequency range from 5 to 256 cycles/image, and the slope of the fitted line was determined (here called *Fourier slope*).

The deviation of the fitted line deviated from the actual Fourier power data (goodness of fit; here called *Fourier sigma*) was expressed as the sum of squares of the deviations of the data points from the fitted line, divided by their number.

### Statistical Analysis

**Figure 2:**
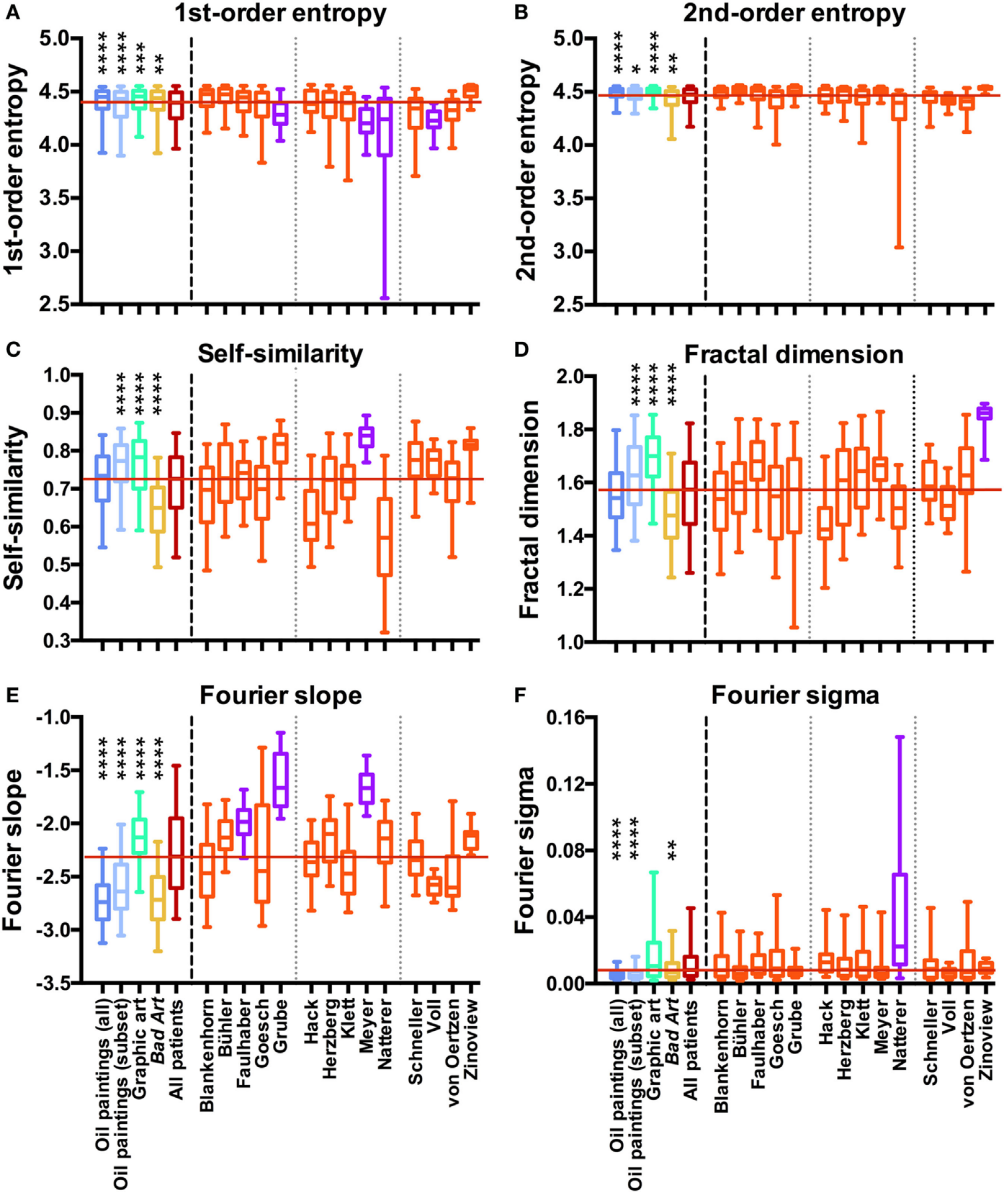
Box plots of first-order entropy **(A)**, second-order entropy **(B)**, self-similarity **(C)**, the fractal dimension **(D)**, Fourier slope **(E)**, and Fourier sigma **(F)** for traditional artworks [*dark blue*, oil paintings (all); *light blue*, oil paintings (1880–1937 subset); *green*, graphic art; *yellow, Bad Art*], for all artists with schizophrenia (*red*) and for individual artists with schizophrenia (*orange* and *pink*), as indicated in **(E,F)**. *Pink* indicates that the median values are outside the overall range of the traditional artworks, as listed in Table 2. Asterisks indicate significant differences between the works by all artists with schizophrenia and the traditional art categories (*p < 0.05; **p < 0.01; ***p < 0.001; ****p < 0.0001). The *red horizontal line* indicates the median value for the artworks by all patients.

Non-parametric tests were used throughout the analysis because the values for most measures were not normally distributed. To analyze the data by non-parametric ANOVAs, we used the Kruskal–Wallis test, followed by Dunn’s post-test. First, we compared grand averages for the five categories of artworks (Western oil paintings, the contemporary subset of the paintings, Western graphic art, *Bad Art*, and the complete data set of art from persons with schizophrenia). Results are displayed in Figure 2 to the left of the vertical dashed lines. Second, separate one-way ANOVAs were carried out for each of the 4 control data sets and the group of 14 sets of paintings by the artists. The significance levels for the differences are indicated by asterisks in Table 2. We calculated effect sizes (*r*) for the differences between the sets of works by each artist and the control data sets from the *z* statistics of a Wilcoxon rank-sum test. Effect sizes are listed in Table 2 for significant differences only. In all analyses, a level of *p* < 0.05 was considered significant. In the box plots (Figure 2), the whiskers bracket 5–95% of the data.

**Table 2:** Effect sizes (*r*) for the differences between the image properties of a given artist and image properties of the control data set of 1,629 oil paintings (O), the subset of 427 oil paintings dating from 1880 to 1937 (S), 200 graphic artworks (G), and 288 pieces of *Bad Art* (B) (Wilcoxon rank-sum test).

	First-order entropy	Second-order entropy	Self-similarity	Fractal dimension	Fourier slope	Fourier sigma
Blankenhorn (*n* = 204)		0.269**** (B)▴	0.129**** (O)▾		0.266**** (O)▴	0.231**** (O)▴ 0.301**** (S)▴
	0.166*** (S)▾		0.385**** (S)▾	0.268**** (S)▾	0.216**** (S)▴
			0.416**** (G)▾	0.512**** (G)▾	0.453**** (G)▾
			0.175** (B)▴	0.179** (B)▴	0.353**** (B)▴

Bühler (*n* = 92)	0.075* (O)▴	0.111*** (O)▴			0.349**** (O)▴
	0.184*** (S)▴	0.222**** (S)▴			0.504**** (S)▴
			0.161* (G)▾	0.330**** (G)▾		0.250**** (G)▾
		0.395**** (B)▴	0.345**** (B)▴	0.320**** (B)▴	0.633****(B)▴

Faulhaber (*n* = 121)				0.211**** (O)▴	0.420**** (O)▴	0.277**** (O)▴
			0.210*** (S)▾	0.153** (S)▴	0.627**** (S)▴	0.408**** (S)▴
			0.266*** (G)▾		0.296** (G)▴
		0.215*** (B)▴	0.408**** (B)▴	0.553**** (B)▴	0.735**** (B)▴	0.217*** (B)▴

Goesch (*n* = 393)	0.092*** (O)▾	0.217**** (O)▾	0.134**** (O)▾		0.329**** (O)▴	0.311**** (O)▴
		0.221**** (S)▾	0.378**** (S)▾	0.256**** (S)▾	0.257**** (S)▴	0.352**** (S)▾
	0.204* (G)▾	0.335**** (G)▾	0.364**** (G)▾	0.425**** (G)▾	0.206**** (G)▾
			0.219**** (B)▴	0.157**** (B)▴	0.364**** (B)▴	0.153*** (B)▴

Grube (*n* = 51)	0.143**** (O)▾		0.166**** (O)▴		0.295**** (O)▴	0.139**** (O)▴
	0.192**** (S)▾		0.183** (S)▴		0.522**** (S)▴	0.208** (S)▴
	0.357**** (G)▾			0.314**** (G)▾	0.602**** (G)▴
	0.279**** (B)▾	0.262**** (B)▴	0.517**** (B)▴	0.150* (B)▴	0.613**** (B)▴

Hack (*n* = 31)			0.131**** (O)▾	0.122**** (O)▾	0.169**** (O)▴	0.171**** (O)▴
			0.316**** (S)▾	0.291**** (S)▾	0.209** (S)▴	0.294**** (S)▴
			0.419**** (G)▾	0.488**** (G)▾	0.290* (G)▾
					0.323**** (B)▴	0.201** (B)▴

Herzberg (*n* = 36)					0.218**** (O)▴	0.121**** (O)▴
			0.154* (S)▾		0.332**** (S)▴	0.195** (S)▴
			0.221* (G)▾	0.211* (G)▾
			0.201** (B)▴	0.227*** (B)▴	0.443****(B)▴

Klett (*n* = 72)	0.075* (O)▾	0.093** (O)▾		0.083* (O)▴	0.182**** (O)▴	0.160**** (O)▴
			0.233**** (S)▾		0.166* (S)▴	0.245**** (S)▴
	0.184* (G)▾	0.275**** (G)▾	0.288*** (G)▾	0.208* (G)▾	0.435**** (G)▾
			0.310**** (B)▴	0.357**** (B)▴	0.302**** (B)▴

Meyer (*n* = 44)	0.178**** (O)▾		0.221**** (O)▴	0.115***(O)▴	0.276****(O)▴	0.083* (O)▴
	0.273**** (S)▾		0.318**** (S)▴		0.495****(S)▴
	0.448**** (G)▾		0.340**** (G)▴		0.591****(G)▴
	0.363**** (B)▾	0.253*** (B)▴	0.563**** (B)▴	0.396****(B)▴	0.583****(B)▴

Natterer (*n* = 32)	0.115**** (O)▾	0.141**** (O)▾	0.162**** (O)▾		0.198**** (O)▴	0.190**** (O)▴
	0.178** (S)▾	0.235**** (S)▾	0.353**** (S)▾	0.223**** (S)▾	0.298**** (S)▴	0.340**** (S)▴
	0.302**** (G)▾	0.399**** (G)▾	0.473**** (G)▾	0.452**** (G)▾		0.253** (G)▴
	0.240**** (B)▾				0.405**** (B)▴	0.323**** (B)▴

Schneller (*n* = 36)	0.093** (O)▾		0.081* (O)▴		0.194**** (O)▴	0.085** (O)▴
					0.253*** (S)▴	0.134* (S)▴
	0.243** (G)▾	0.266** (G)▾		0.279* (G)▾
	0.024* (B)▾		0.389**** (B)▴	0.294****(B)▴	0.373**** (B)▴

Voll (*n* = 102)	0.269**** (O)▾	0.247**** (O)▾	0.130**** (O)▴	0.087** (O)▾	0.178**** (O)▴	0.105** (O)▴
	0.373**** (S)▾	0.350**** (S)▾		0.310**** (S)▾
	0.565**** (G)▾	0.583**** (G)▾		0.606**** (G)▾	0.705**** (G)▾	0.278**** (G)▾
	0.481**** (B)▾	0.184** (B)▴	0.588**** (B)▴

von Oertzen (*n* = 26)	0.090** (O)▾	0.130**** (O)▾			0.101** (O)▴	0.065* (O)▴
		0.214*** (S)▾
	0.243* (G)▾	0.378**** (G)▾			0.351**** (G)▾
	0.184* (B)▾		0.209* (B)▴	0.266****(B)▴

von Zinoview (*n* = 16)	0.080* (O)▴	0.106*** (O)▴	0.114*** (O)▴	0.162**** (O)▴	0.158**** (O)▴	0.103** (O)▴
	0.172** (S)▴	0.206*** (S)▴		0.282**** (S)▴	0.257**** (S)▴	0.173* (S)▴
				0.383** (G)▴
	0.184* (B)▴	0.307**** (B)▴	0.347**** (B)▴	0.379**** (B)▴	0.343**** (B)▴

*The bold letters indicate cases, in which values for an artist are above or below the values for each of the four control datasets. The significance levels (*p*) were calculated with an ANOVA (Dunn’s post-test). Cohen (38) reported the following intervals for *r*: 0.1–0.3, small effect; 0.3–0.5, intermediate effect; larger than 0.5, strong effect*.

*n, number of images; ▾, ▴, mean values for the artist’s images are below or above the values of the control dataset, respectively*.

*p < 0.05; **p < 0.01; ***p < 0.001; ****p < 0.0001.

## Results

We measured six statistical image properties in the works of 14 artists who are featured in the Prinzhorn Collection. Figure 2 (left-hand side of the dashed line) displays the results for a comparison of grand average values for the control data sets of traditional prestigious artworks (oil paintings, the contemporary subset of the oil paintings, and graphic art), *Bad Art* and artworks by patients with schizophrenia. Average results differed significantly between the five image categories (Kruskal–Wallis test; *df* = 4 and *p* < 0.0001 for all measures; first-order entropy of edge orientations, *H* = 78; second-order entropy, *H* = 112; self-similarity, *H* = 301; fractal dimension, *H* = 285; Fourier slope, *H* = 1099; and Fourier sigma, *H* = 497). However, the average values for the artworks by the artists with schizophrenia were within the overall range of the control data sets for all image properties (Figures 2B–F), except for first-order entropy, which was lower in the patients’ works than in each of the three control categories (Figure 2A; Dunn’s post-test).

As expected, results also differed between the artists with schizophrenia (Kruskal–Wallis test) for all image properties (*df* = 13 and *p* < 0.0001 for all image properties; first-order entropy, *H* = 236; second-order entropy, *H* = 228; self-similarity, *H* = 314; fractal dimension, *H* = 201; Fourier slope, *H* = 389; and Fourier sigma, *H* = 88).

We next compared the images of individual artists with each of four control data sets (for box plots of the results, see Figure 2, right-hand side of the dashed line). Table 2 lists the effect sizes and significance levels for the significant differences between the mean image properties for each individual artist and each of the four traditional art (control) categories.

**Figure 3:**
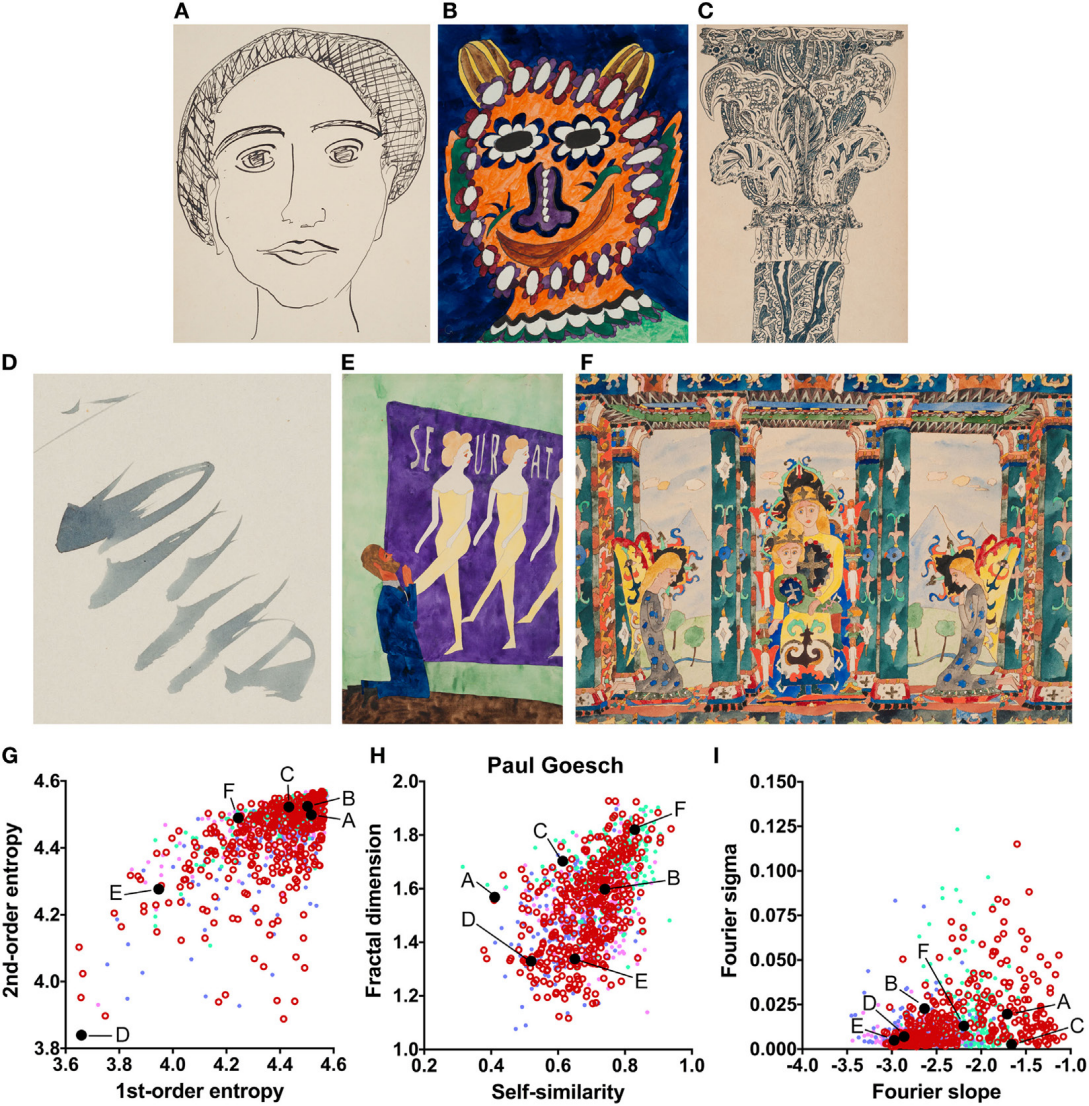
Exemplary images **(A–F)** and results for the measured image properties [**(G)** first- and second-order entropy; **(H)** self-similarity and fractal dimension; **(I)** Fourier slope and sigma] for the artist Paul Goesch. In the dot plots, each dot represents one image (*pink dots*, oil paintings; *green dots*, graphic art; *light blue dots, Bad Art; red open circles*, artist). The letters and the black dots in **(G–I)** indicate the values of the exemplary images **(A–F)**. Reproduced with permission, © Sammlung Prinzhorn, Universitätsklinikum Heidelberg.

The results indicate that the artworks of eight artists with schizophrenia possess image properties that are well within the range of the traditional art categories and *Bad Art* (Else Blankenhorn, Franz Karl Bühler, Paul Goesch, Heinrich Hack, Oskar Herzberg and August Klett, Joseph Schneller, and Clemens von Oertzen). For example, Figure 3 illustrates results for the artworks by Paul Goesch, whose large oeuvre of 393 artworks is rather diverse in terms of their subjective impression on the beholder. We note that his works also differ in how much they resemble traditional artworks. For example, the image in Figure 3B shows values well within the traditional art range while the image in Figure 3D deviates more strongly in all six values. The other works in Figure 3 deviate in some values only Figures 3G–I. For example, the images in Figures 3C,F have relatively high values for the fractal dimension and the Fourier slope; the artwork in Figure 3E lacks an even distribution of edge orientations (low first- and second-order entropies). Results for the other artists, whose works are similar to the traditional art data sets, are shown in Figures S2–S8 in Supplementary Material.

**Figure 4:**
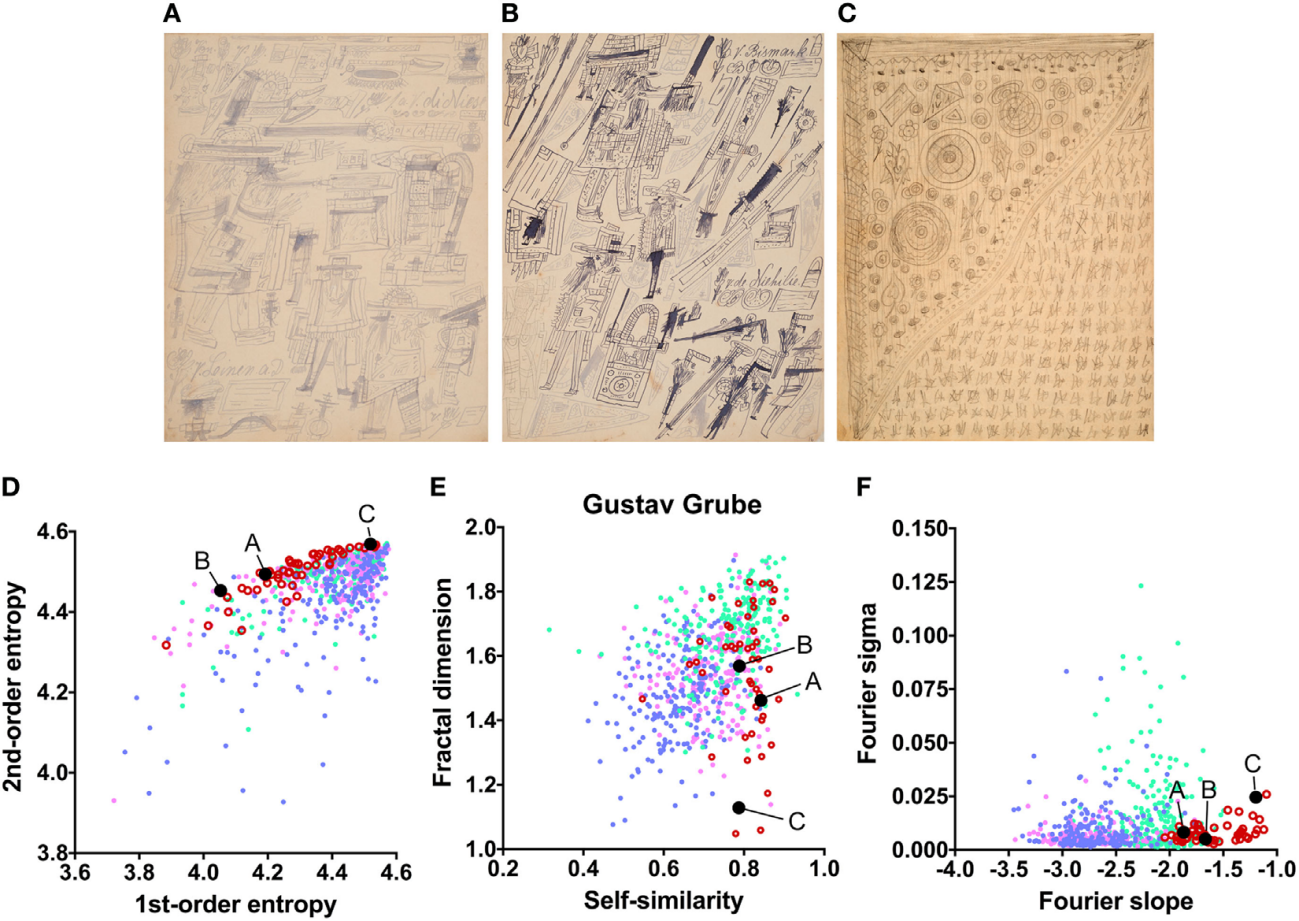
Exemplary images **(A–C)** and results for the measured image properties [**(D)** first- and second-order entropy; **(E)** self-similarity and fractal dimension; **(F)** Fourier slope and sigma] for the artist Gustav Grube. In the dot plots, each dot represents one image (*pink dots*, oil paintings; *green dots*, graphic art; *light blue dots, Bad Art; red open circles*, artist). The letters and the black dots in **(D–F)** indicate the values of the exemplary images **(A–C)**. The image in **(C)** was enhanced in contrast to improve the visibility of its pictorial structure. Reproduced with permission, © Sammlung Prinzhorn, Universitätsklinikum Heidelberg.

**Figure 5:**
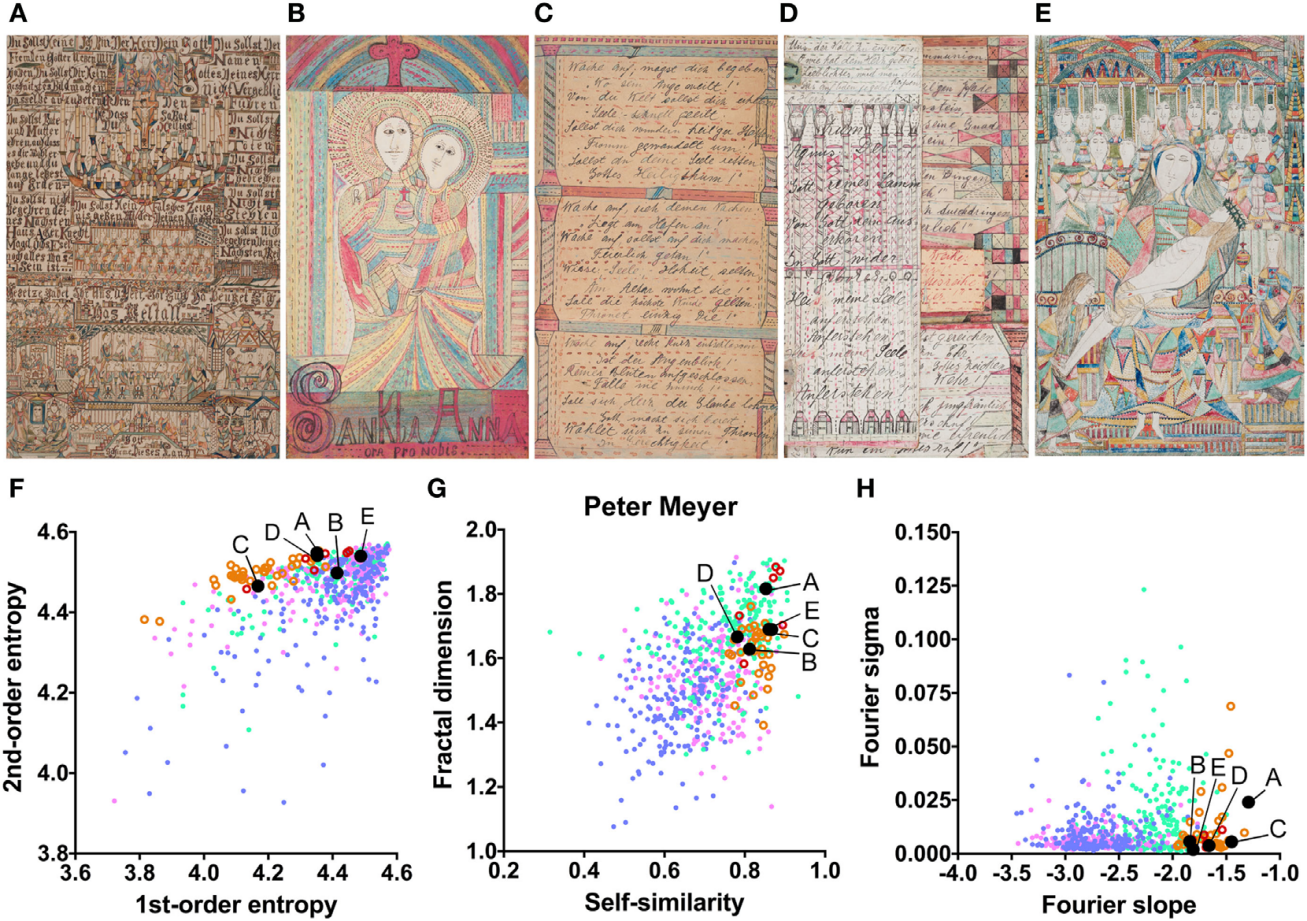
Exemplary images **(A–E)** and results for the measured image properties [**(F)** first- and second-order entropy; **(G)** self-similarity and fractal dimension; **(H)** Fourier slope and sigma] for the artist Peter Meyer. In the dot plots, each dot represents one image; *pink dots*, oil paintings; *green dots*, graphic art; *light blue dots, Bad Art*; *red open circles*, drawings by the artist [examples in **(B,E)**]; *orange open circles*, text images by the artist [examples in **(A,C,D)**]. The letters and the black dots in (**F–H)** indicate the values of the exemplary images shown in (**A–E)**. Reproduced with permission, © Sammlung Prinzhorn, Universitätsklinikum Heidelberg.

**Figure 6:**
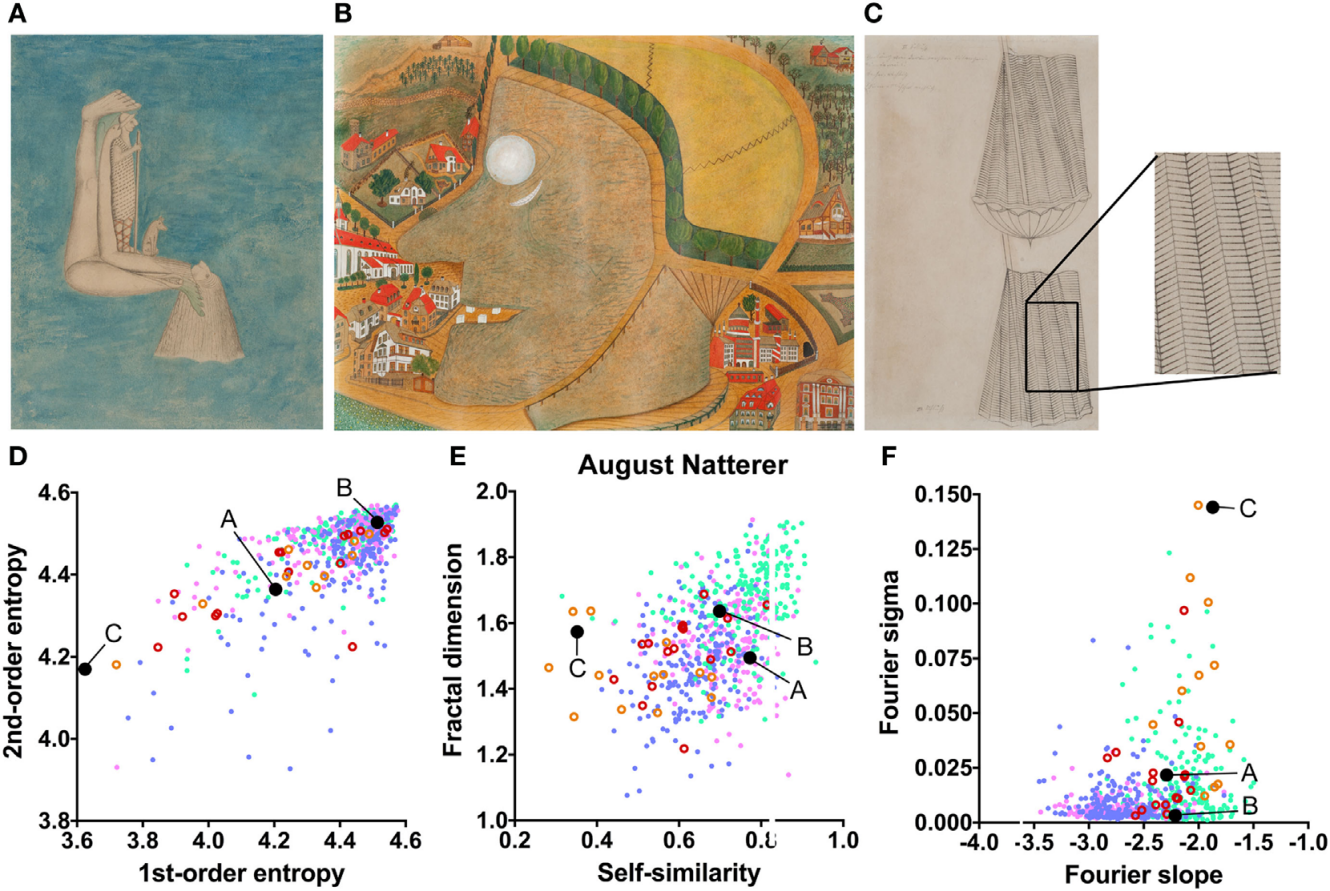
Exemplary images **(A–C)** and results for the measured image properties [**(D)** first- and second-order entropy; **(E)** self-similarity and fractal dimension; **(F)** Fourier slope and sigma] for the artist August Natterer. In the dot plots, each dot represents one image [*pink dots*, oil paintings; *green dots*, graphic art; *light blue dots, Bad Art*; *red open circles*, drawings by the artist [examples in **(A,B)**]; *orange open circles*, sketches by the artist [example in **(C)**]]. The letters and the black dots in (**D–F)** indicate the values of the exemplary images **(A–C)**. The area boxed in **(C)** is shown at a higher magnification to the right of the panel. Reproduced with permission, © Sammlung Prinzhorn, Universitätsklinikum Heidelberg.

**Figure 7:**
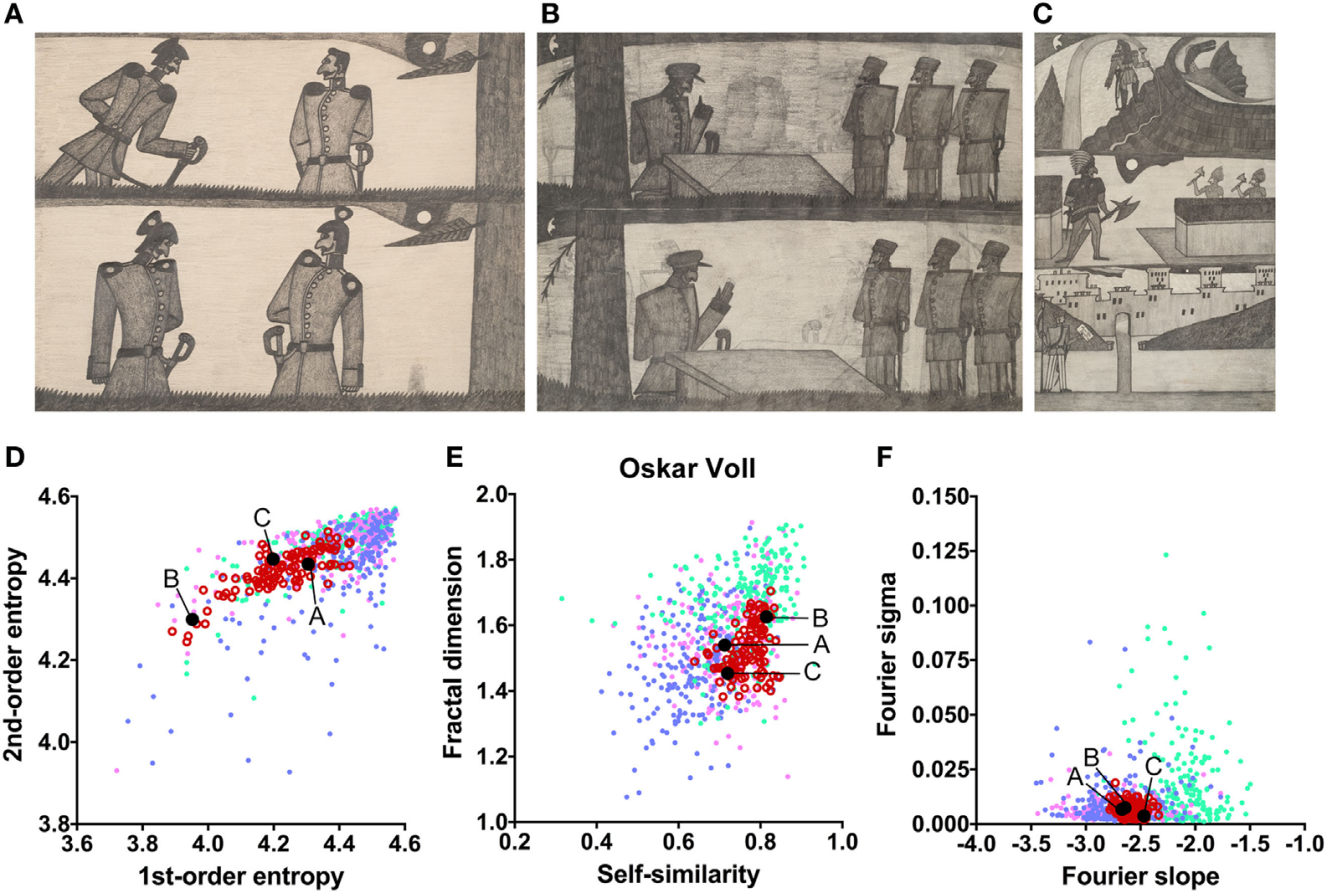
Exemplary images **(A–C)** and results for the measured image properties [**(D)** first- and second-order entropy; **(E)** self-similarity and fractal dimension; **(F)** Fourier slope and sigma] for the artist Oskar Voll. In the dot plots, each dot represents one image (*pink dots*, oil paintings; *green dots*, graphic art; *light blue dots, Bad Art*; *red open circles*, artist). The letters and the black dots in (**D–F)** indicate the values of the exemplary images **(A–C)**. Reproduced with permission, © Sammlung Prinzhorn, Universitätsklinikum Heidelberg.

**Figure 8:**
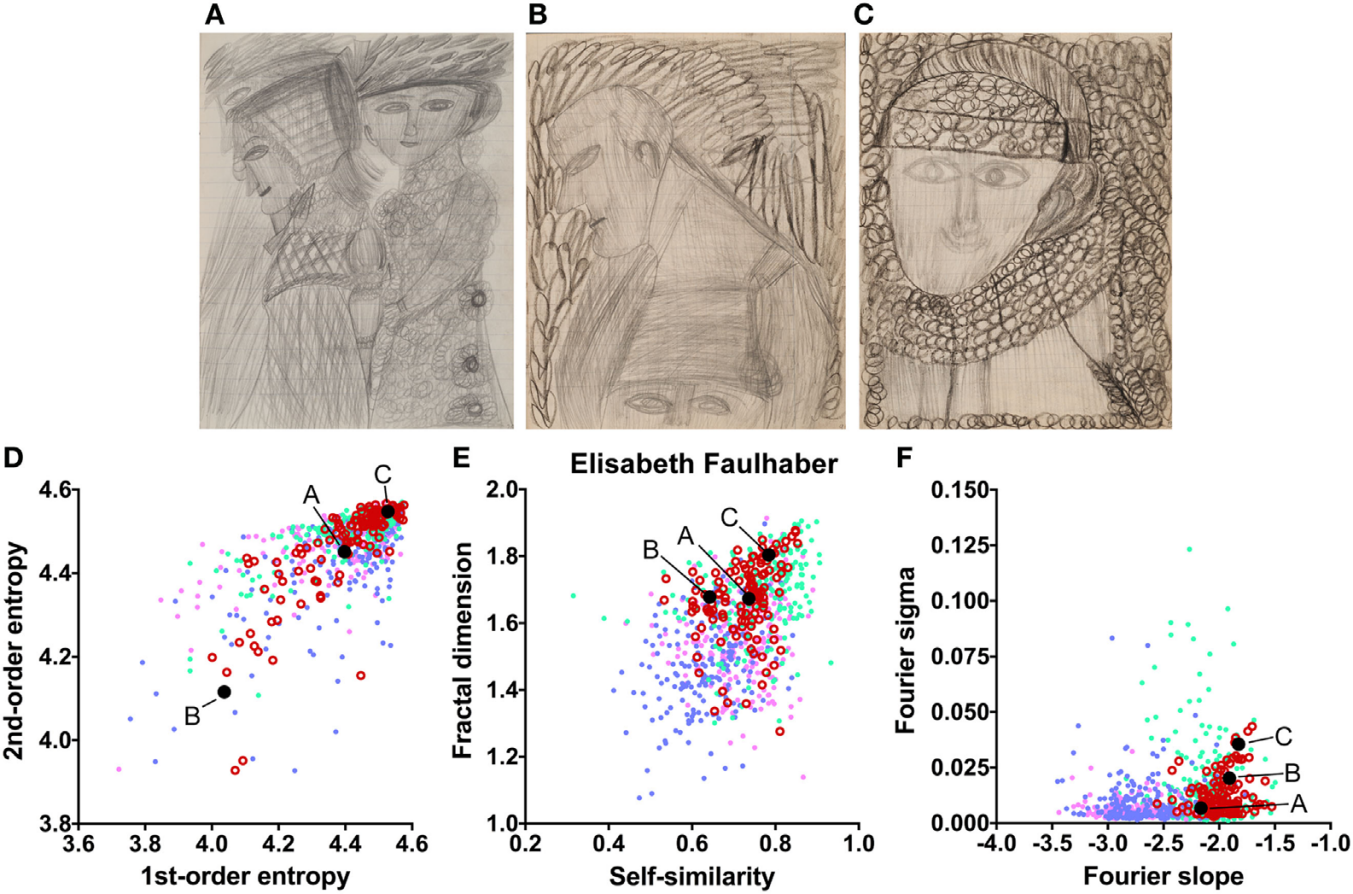
Exemplary images **(A–C)** and results for the measured image properties [**(D)** first- and second-order entropy; **(E)** self-similarity and fractal dimension; **(F)** Fourier slope and sigma] for the artist Elisabeth Faulhaber. In the dot plots, each dot represents one image (*pink dots*, oil paintings; *green dots*, graphic art; *light blue dots, Bad Art*; *red open circles*, artist). The letters and the black dots in **(D–F)** indicate the values of the exemplary images **(A–C)**. Reproduced with permission, © Sammlung Prinzhorn, Universitätsklinikum Heidelberg.

**Figure 9:**
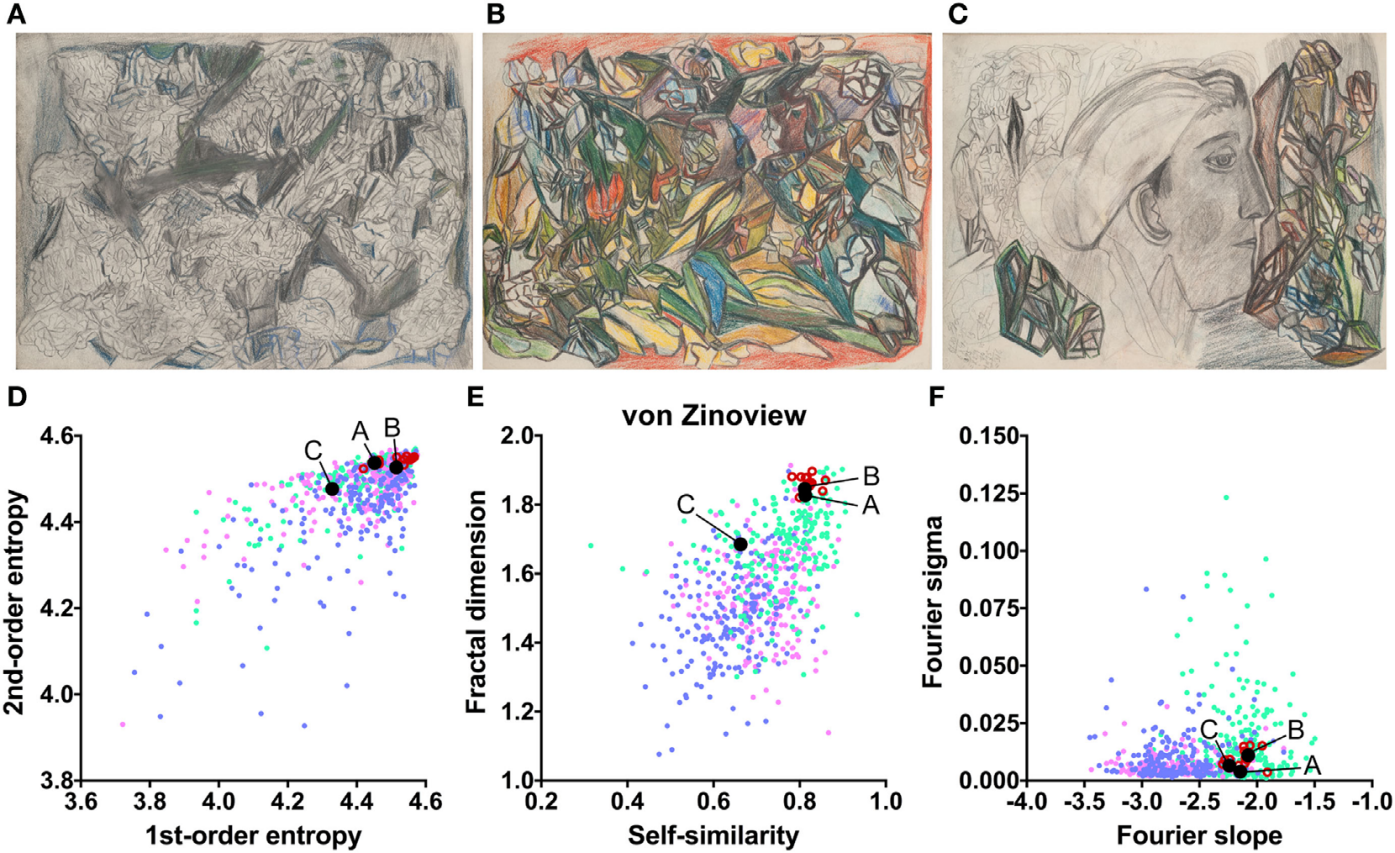
Exemplary images **(A–C)** and results for the measured image properties [**(D)** first- and second-order entropy; **(E)** self-similarity and fractal dimension; **(F)** Fourier slope and sigma] for the artist von Zinoview. In the dot plots, each dot represents one image (*pink dots*, oil paintings; *green dots*, graphic art; *light blue dots, Bad Art*; *red open circles*, artist). The letters and the black dots in **(D–F)** indicate the values of the exemplary images **(A–C)**. Reproduced with permission, © Sammlung Prinzhorn, Universitätsklinikum Heidelberg.

For six artists with schizophrenia, we found that the values for one or more image properties deviated systematically from the range of the four control data sets (Figure 2; Table 2), i.e., for a given measure, the average values for the patient were higher or lower than the values for each of the four control data sets. Representative results for these six artists are illustrated in Figures 4–9.

The mean values for the monochrome works by Gustav Grube (Figure 4; Table 2) deviate from the control art categories in their first-order entropy and the Fourier slope. The lower first-order entropy reflects a predominance of oblique orientations in his artworks, which result in uneven orientation histograms (for example, see Figures 4A,D). The higher (less negative) Fourier slope indicates a larger proportion of high spatial frequencies, due to the relatively large amount of fine detail in his works, especially in the images that depict repetitive pictorial elements (Figures 4C,F). Values for the other measures are well within the range of traditional artworks. For example, the even distribution of the pictorial elements across the images results in a high self-similarity. We observed similar deviances in the more colorful works by Peter Meyer (Figure 5; Table 2). Particular (horizontal and vertical) orientations are predominant in his drawings (low first-order entropy; Figures 5C,F). Moreover, many of Peter Meyer’s works are characterized by a large amount of fine detail (high Fourier slope; Figures 5A,C,H). In addition, similar pictorial detail is distributed evenly across the images (high self-similarity; Figures 5A,C,E,G).

In the data set of August Natterer’s works (Figure 6A–C; Table 2), several drawings are composed of repetitive line elements of similar orientations (low first-order entropy; see enlarged detail in Figure 6C) and specific spatial frequencies that dominate the images (high Fourier sigma; orange circles in Figure 6D–F).

The works of three other artists deviate in one measure only (Table 2). The drawings by Oskar Voll exhibit an uneven distribution of edge orientations (low first-order entropy; Figure 7). Elisabeth Faulhaber (Figure 8) created many monochrome drawings with a large amount of fine detail, at the expense of global structure (high Fourier slope). Most of the drawings by Frau von Zinoview (Figure 9) appear rather complex (high fractal dimension); for the other measures, they yielded values that did not differ from the control data sets, in particular, from monochrome graphic art.

Interestingly, the mean values for second-order entropy are within the range of traditional artworks and *Bad Art* for all artists.

## Discussion

This study represents the first large-scale, computer-based analysis of the works by artists who were diagnosed with dementia praecox or schizophrenia (except for Elisabeth Faulhaber and Gustav Grube, whose diagnoses are unknown). It has to be pointed out that, at the time when the diagnoses were made, rigorous criteria were not yet applied, as it is done today. Thus, some uncertainty must be taken into account with regard to the diagnoses. In addition, we do not have any information on possible co-morbidities in the patients, which limits the interpretability of our data further. Nevertheless, our study demonstrates that it is feasible to analyze formal aspects of works by artists with schizophrenia by objective scientific means and to compare the results with those of artworks by healthy artists. This scientific approach may supplement the many art–historical efforts that tackle the question of what effect (if any) schizophrenia has on the artworks of patients suffering from the disease. A similar approach has already contributed to our understanding of the aesthetic preferences of patients with other types of mental or brain disease, such as dementia and cerebral insults (18, 39–44).

To identify image properties that diverge between artists with schizophrenia and healthy artists, we compared the works of artists with schizophrenia with data sets of traditional artworks of Western provenance. Two of the data sets were from prestigious museums and included mostly colored representational oil paintings and monochrome works on paper (graphic art), respectively, from the 16th to the 20th century. From the set of oil paintings, we extracted the subset of paintings that were created between 1880 and 1937. This control data set may resemble the patients’ artworks more closely in artistic style than the complete set of oil paintings. A fourth database included modern artworks of lesser importance (so-called *Bad Art*; see Introduction) and included works by lesser-known artists, most of whom had not received formal artistic training. Together, the four databases are similar in style and technique to the artworks by persons with schizophrenia. In particular, the works by Elisabeth Faulhaber, Gustav Grube, Oskar Voll, and Frau von Zinoview, which were less colorful than the works by the other artists, resembled the control set of monochrome graphic art. Image properties were considered deviating if they were lower or higher than each of the four control databases, i.e., outside their overall range (Table 2).

The works by 8 of the 14 artists with schizophrenia (57%) were more or less within the range of traditional artworks, i.e., they do not differ from one or more of the control data sets (Table 2). Our results thus support the notion that there is a large overlap between art by patients with schizophrenia who created artworks and by mentally healthy artists (7).

The works of only six of the artists showed formal characteristics that deviate markedly from the artworks by healthy artists. Two of the patients (Gustav Grube and Peter Meyer; Figures 4 and 5) showed a similar pattern of differences. In the works of both artists, there is a large amount of fine detail relative to coarse image structure (high Fourier slope), confirming previous results by Graham and Meng (17) in 12 works by 5 artists with schizophrenia. Moreover, particular edge orientations predominate (low first-order entropy), and self-similarity is relatively high in their artworks, although self-similarity does not exceed the range of graphic art for Gustav Grube’s drawings. Together, these properties are likely to relate to the subjective feeling that their works are filled with a vast amount of small detail (high Fourier slope) in every corner of the image (high self-similarity), an impression that may reflect the “horror vacui” [see Introduction (12)]. We observed low first-order entropy in the works by two other artists. In particular, the drawings by Oskar Voll appear rigid due to the excess of cardinal orientations (Figure 7), and some of the pencil drawings by August Natterer (Figure 6C) display hatchings of repetitive lines of a restricted range of orientations, as found in technical drawings. These regularly spaced line elements translate into a relative dominance of particular spatial frequencies that, in turn, cause deviations from the straight fitted lines in the Fourier plot (high Fourier sigma). In contrast to first-order entropy, second-order entropy appeared well within the range of the control data sets for all artists with schizophrenia.

This study is exploratory, and any reference to visual mechanisms in schizophrenia (45–48) that may possibly underlie the observed differences to healthy artists is highly speculative. In particular, a deficit in the perception of global image structure may cause artists with schizophrenia to reduce coarse (global) structure (low spatial frequencies) relative to local detail (high spatial frequencies) in their creations, as previously proposed by Graham and Meng (17). Such a shift to fine image detail may lead to a shallower Fourier slope, which was indeed observed for three of the patients in this study (Table 2). Whether the other observed differences relate to the type of defects in perceptual organization that have been described in patients with schizophrenia (49, 50) remains to be studied in the future.

It should be emphasized that the present investigation was restricted to formal aspects of image composition, and we did not consider image content. Other studies suggest that it is the content of artworks that reflects the pathological mental state of artists with schizophrenia [e.g., delusions, erotic content (7, 12–14)]. In our data set of artworks, we did not find many examples of content that reflected an obviously pathological mental state. However, we cannot exclude the possibility that there are associations between image content and form in our study. For example, it is possible that artists with particular types of delusions may chose to produce drawings with specific content, which, in turn, is linked to particular image properties. Another feature of art by persons with schizophrenia relates to their special imagination and creativity. These aspects must be evaluated in the art–historical context (3, 51) and are beyond the scope of the present study.

In conclusion, by introducing a set of objective image properties, our study opens novel perspectives for the scientific investigation of visual artworks created by persons who suffer from schizophrenia. Future studies may investigate the relation between the image properties and different types of schizophrenia, and the effect of disease exacerbations and medication. Eventually, when combined with medical and art–historical analyses, such studies may help us to understand what is special about schizophrenia and its puzzling relation to human visual perception and creativity. The present study shows that an objective quantification of the extent, to which artworks by patients with schizophrenia differ from works by healthy artists, is possible by measuring statistical image properties in large sets of artworks.

## Author Contributions

GH carried out the experiments and analyzed the data. GH and CR conceived study and wrote the manuscript. AB contributed computer code and calculations. CR supervised the study at all stages.

## Conflict of Interest Statement

The authors declare that the research was conducted in the absence of any commercial or financial relationships that could be construed as a potential conflict of interest.

## Footnotes

^1^http://prinzhorn.ukl-hd.de.

^2^http://officialbadartmuseumofart.com.
