# Supplementary material for: Statistical Image Properties in Works from the Prinzhorn Collection of Artists with Schizophrenia
Source: Front Psychiatry. 2017 Dec 11;8:273. doi: 10.3389/fpsyt.2017.00273 (PMC5732538; doi:10.3389/fpsyt.2017.00273)
Supplement: Supplementary file 1 [file image_1.pdf]

Supplementary Figure 1

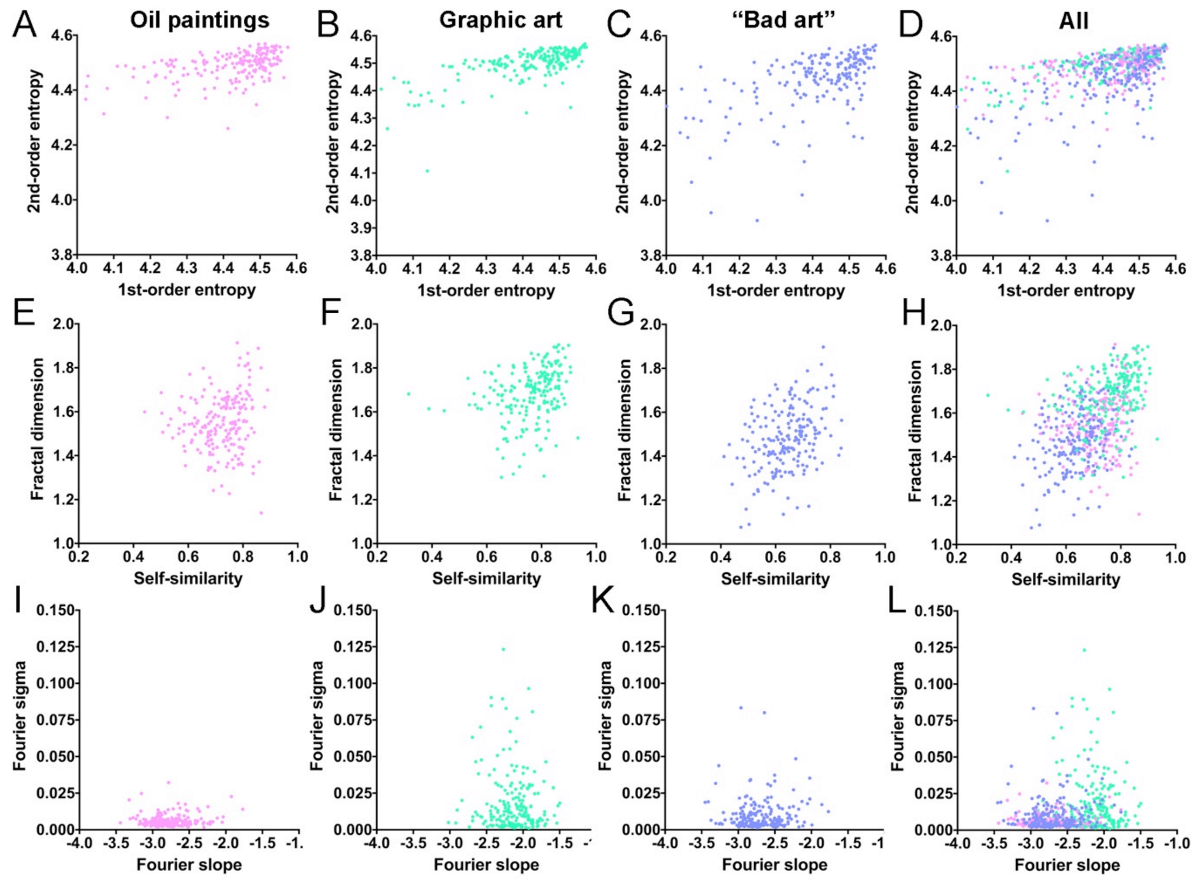

Supplementary Figure 1. Results for the reference (control) datasets of traditional art (oil paintings [A,E,I], graphic art [B,F,J], and *Bad Art* [C,G,K]) for the measured image properties (first-order and second-order entropy [A,B,C,D]; self-similarity and fractal dimension [E,F,G,H]; and Fourier slope and sigma [I,J,K,L]). All three image properties are plotted together in (D,H,L). Each dot represents one image. For each image category, 200 randomly selected example images are plotted. The data are from the study by Redies and Brachmann (2017).

Supplementary Figure 2

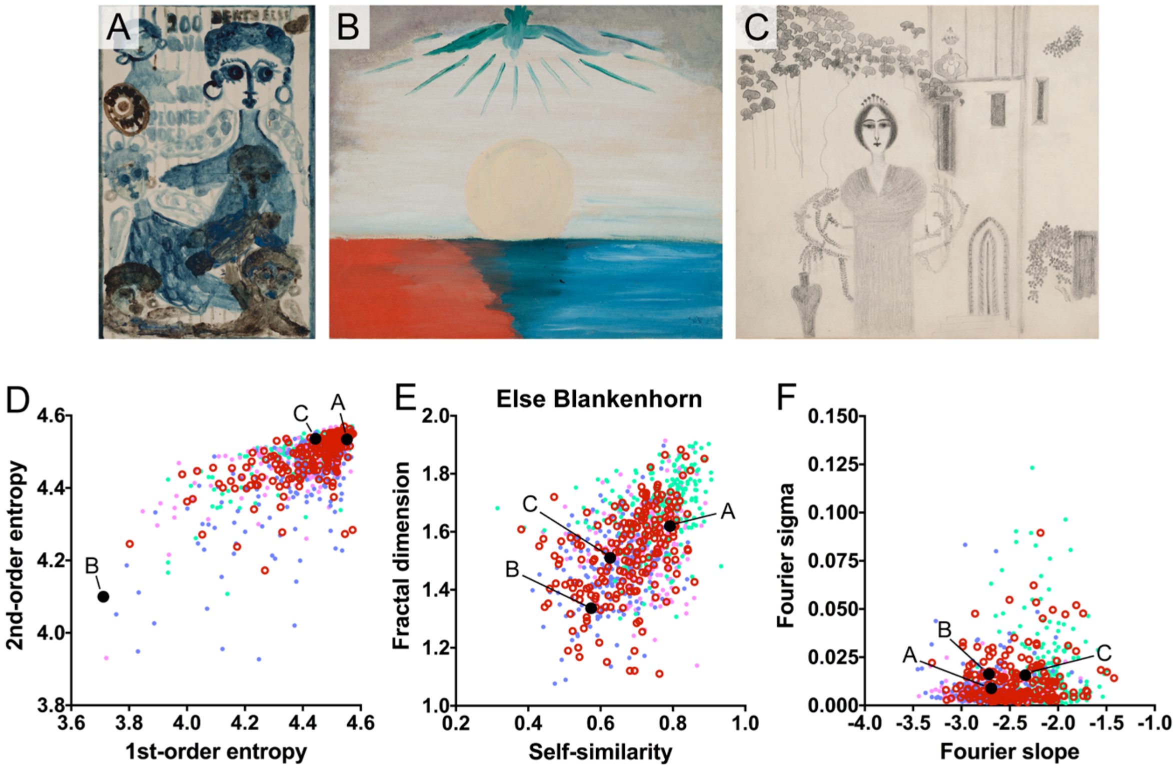

Supplementary Figure 2. Exemplary images (A-C) and results for the measured image properties (D, first-order and second-order entropy; E, self-similarity and fractal dimension; F, Fourier slope and sigma) for the artist Else Blankenhorn. In the dot plots, each dot represents one image (*pink dots*, oil paintings; *green dots*, graphic art; *light blue dots*, *Bad Art; red open circles,* artist). The letters and the black dots in D-F indicate the values of the exemplary images (A-C). Reproduced with permission, © Sammlung Prinzhorn, Universitätsklinikum Heidelberg.

Supplementary Figure 3

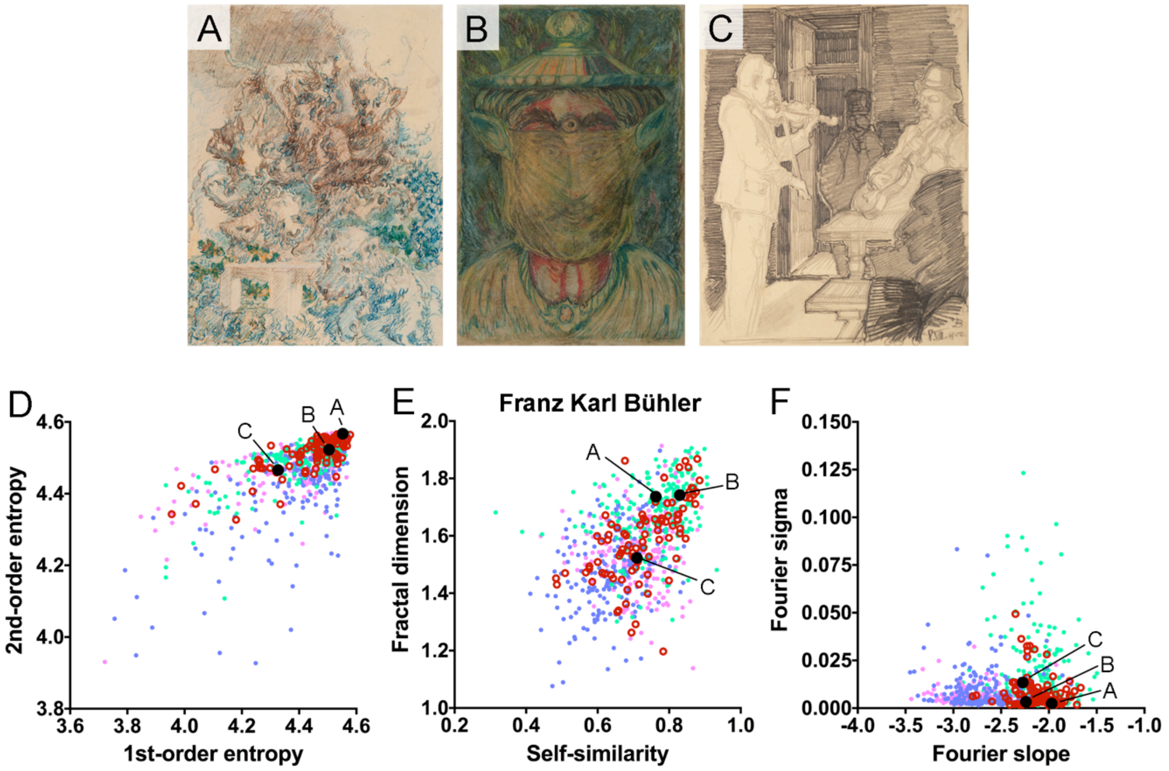

Supplementary Figure 3. Exemplary images (A-C) and results for the measured image properties (D, first-order and second-order entropy; E, self-similarity and fractal dimension; F, Fourier slope and sigma) for the artist Franz Karl Bühler. In the dot plots, each dot represents one image (*pink dots*, oil paintings; *green dots*, graphic art; *light blue dots*, *Bad Art; red open circles,* artist). The letters and the black dots in D-F indicate the values of the exemplary images (A-C). Reproduced with permission, © Sammlung Prinzhorn, Universitätsklinikum Heidelberg.

Supplementary Figure 4

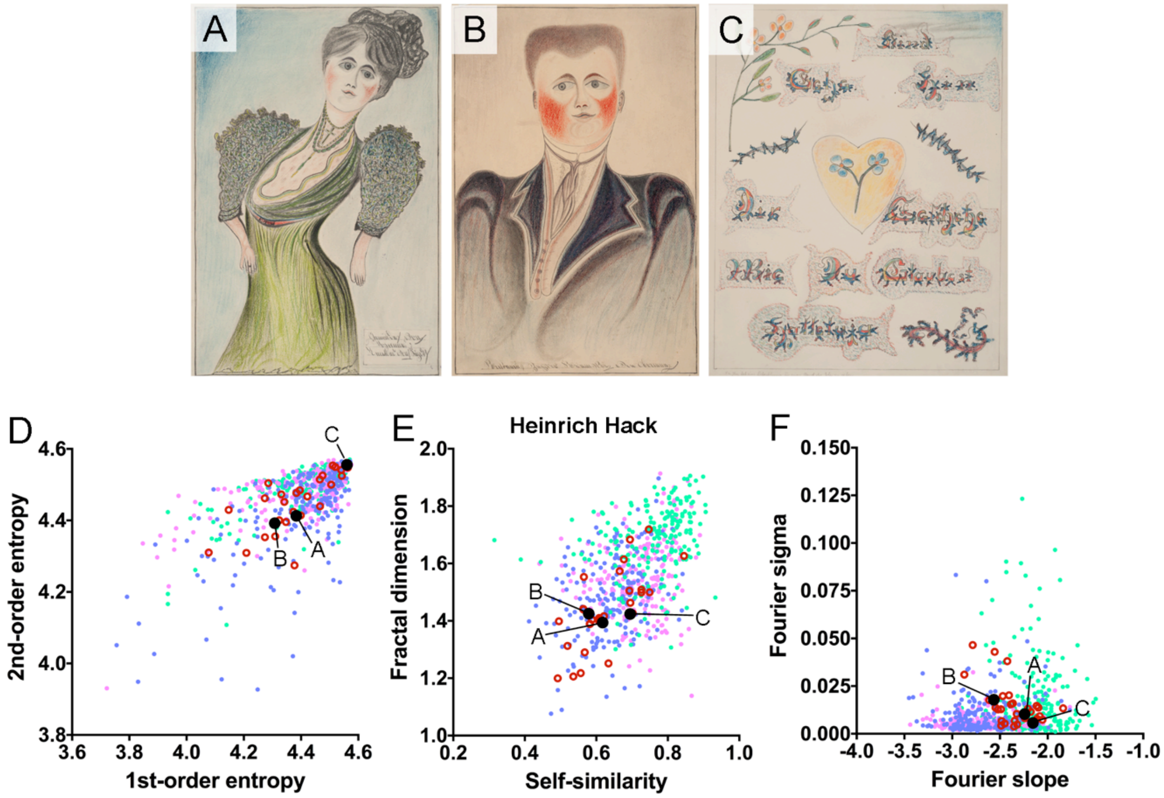

Supplementary Figure 4. Exemplary images (A-C) and results for the measured image properties (D, first-order and second-order entropy; E, self-similarity and fractal dimension; F, Fourier slope and sigma) for the artist Heinrich Hack. In the dot plots, each dot represents one image (*pink dots*, oil paintings; *green dots*, graphic art; *light blue dots*, *Bad Art; red open circles,* artist). The letters and the black dots in D-F indicate the values of the exemplary images (A-C). Reproduced with permission, © Sammlung Prinzhorn, Universitätsklinikum Heidelberg.

Supplementary Figure 5

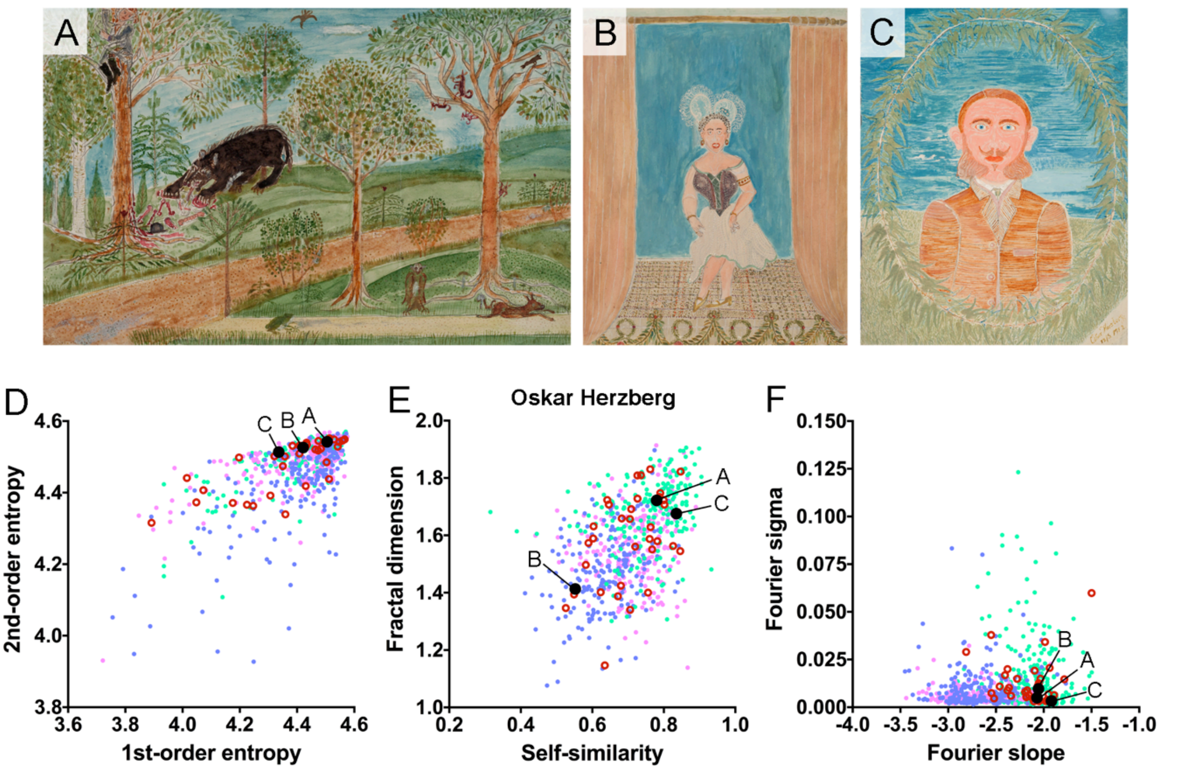

Supplementary Figure 5. Exemplary images (A-C) and results for the measured image properties (D, first-order and second-order entropy; E, self-similarity and fractal dimension; F, Fourier slope and sigma) for the artist Oskar Herzberg. In the dot plots, each dot represents one image (*pink dots*, oil paintings; *green dots*, graphic art; *light blue dots*, *Bad Art; red open circles,* artist). The letters and the black dots in D-F indicate the values of the exemplary images (A-C). Reproduced with permission, © Sammlung Prinzhorn, Universitätsklinikum Heidelberg.

Supplementary Figure 6

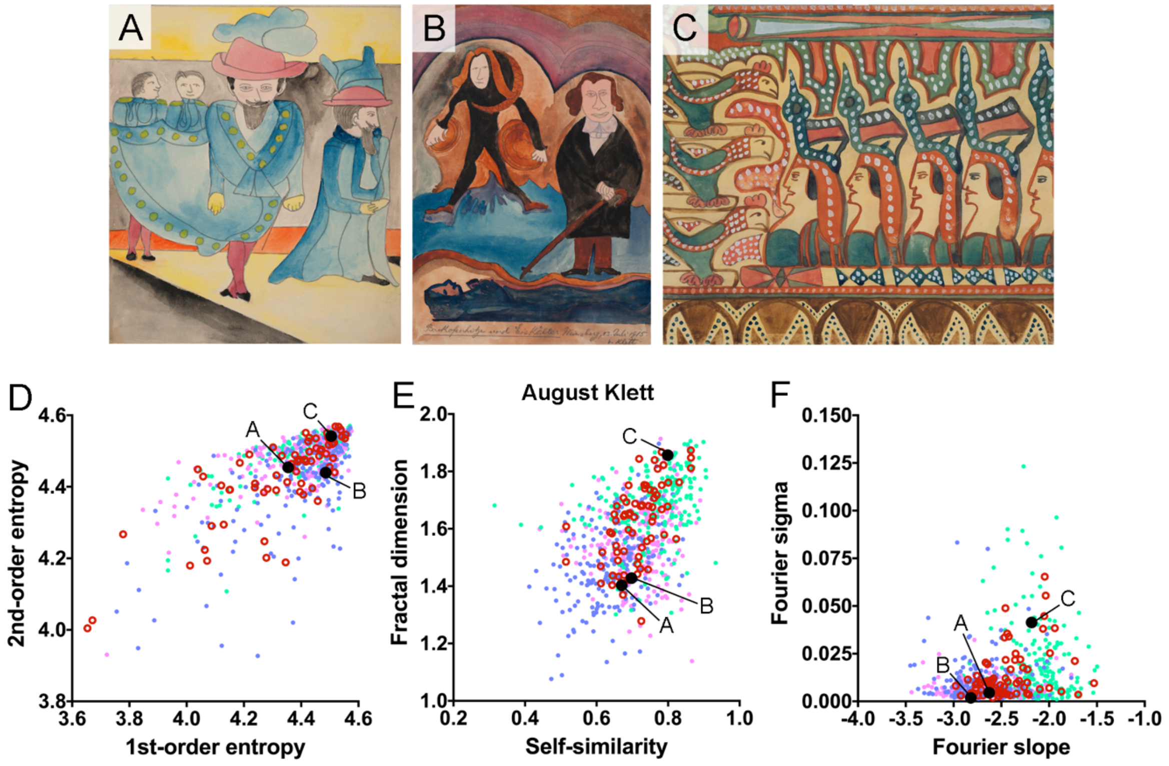

Supplementary Figure 6. Exemplary images (A-C) and results for the measured image properties (D, first-order and second-order entropy; E, self-similarity and fractal dimension; F, Fourier slope and sigma) for the artist August Klett. In the dot plots, each dot represents one image (*pink dots*, oil paintings; *green dots*, graphic art; *light blue dots*, *Bad Art; red open circles,* artist). The letters and the black dots in D-F indicate the values of the exemplary images (A-C). Reproduced with permission, © Sammlung Prinzhorn, Universitätsklinikum Heidelberg.

Supplementary Figure 7

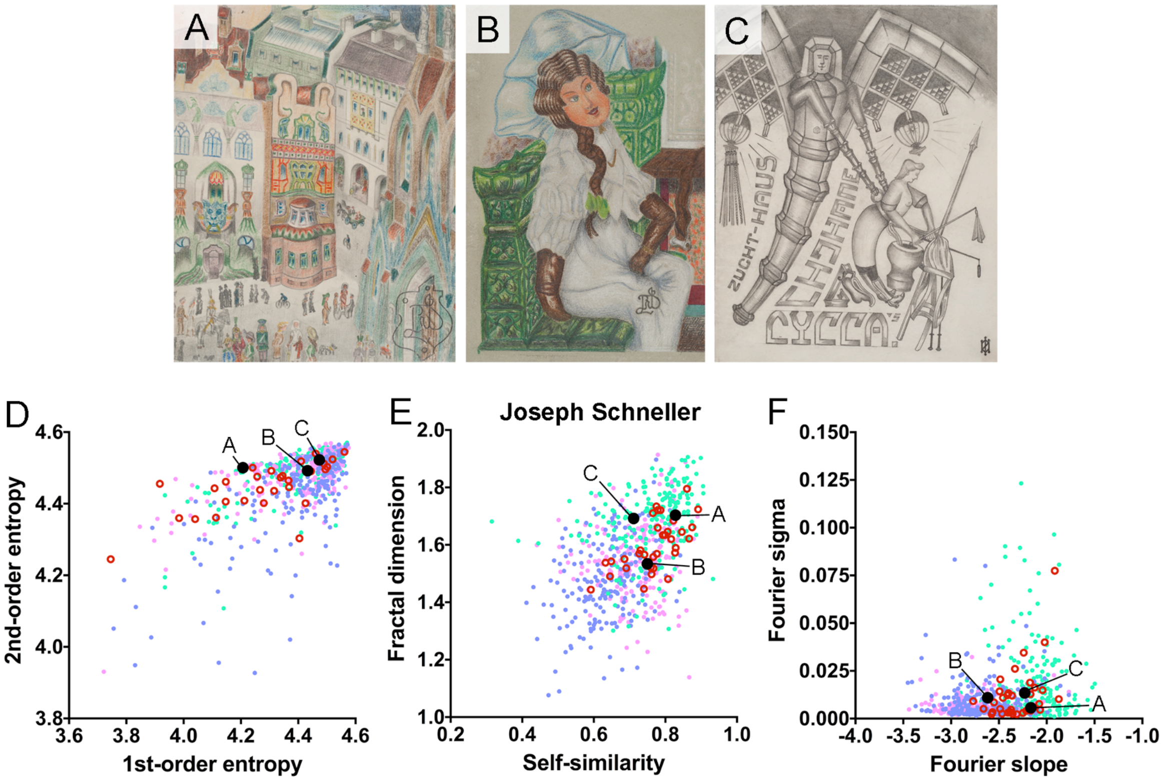

Supplementary Figure 7. Exemplary images (A-C) and results for the measured image properties (D, first-order and second-order entropy; E, self-similarity and fractal dimension; F, Fourier slope and sigma) for the artist Joseph Schneller. In the dot plots, each dot represents one image (*pink dots*, oil paintings; *green dots*, graphic art; *light blue dots*, *Bad Art; red open circles,* artist). The letters and the black dots in D-F indicate the values of the exemplary images (A-C). Reproduced with permission, © Sammlung Prinzhorn, Universitätsklinikum Heidelberg.

Supplementary Figure 8

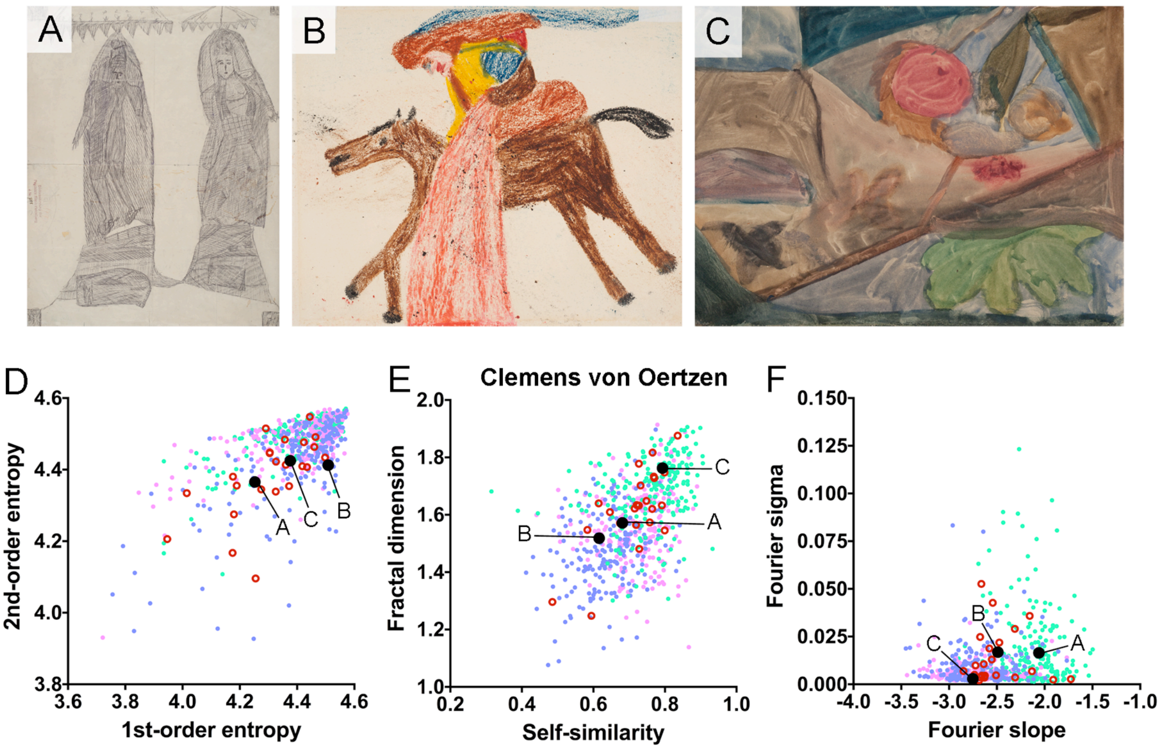

Supplementary Figure 8. Exemplary images (A-C) and results for the measured image properties (D, first-order and second-order entropy; E, self-similarity and fractal dimension; F, Fourier slope and sigma) for the artist Clemens von Oertzen. In the dot plots, each dot represents one image (*pink dots*, oil paintings; *green dots*, graphic art; *light blue dots*, *Bad Art; red open circles,* artist). The letters and the black dots in D-F indicate the values of the exemplary images (A-C). Reproduced with permission, © Sammlung Prinzhorn, Universitätsklinikum Heidelberg.
